# Population antibody responses following COVID-19 vaccination in 212,102 individuals

**DOI:** 10.1038/s41467-022-28527-x

**Published:** 2022-02-16

**Authors:** Helen Ward, Matthew Whitaker, Barnaby Flower, Sonja N. Tang, Christina Atchison, Ara Darzi, Christl A. Donnelly, Alexandra Cann, Peter J. Diggle, Deborah Ashby, Steven Riley, Wendy S. Barclay, Paul Elliott, Graham S. Cooke

**Affiliations:** 1 School of Public Health, Imperial College London, London, UK; 2 MRC Centre for Global Infectious Disease Analysis and Abdul Latif Jameel Institute for Disease and Emergency Analytics, Imperial College London, London, UK; 3 Imperial College Healthcare NHS Trust, London, UK; 4 National Institute for Health Research Imperial Biomedical Research Centre, London, UK; 5 Department of Infectious Disease, Imperial College London, London, UK; 6 Institute of Global Health Innovation at Imperial College London, London, UK; 7 Department of Statistics, University of Oxford, London, UK; 8 CHICAS, Lancaster Medical School, Lancaster University, Lancaster, UK; 9 MRC Centre for Environment and Health, School of Public Health, Imperial College London, London, UK; 10 Health Data Research (HDR) UK London at Imperial College London, London, UK; 11 UK Dementia Research Institute at Imperial College London, London, UK

**Keywords:** Viral infection, SARS-CoV-2, Epidemiology

## Abstract

Population antibody surveillance helps track immune responses to COVID-19 vaccinations at scale, and identify host factors that may affect antibody production. We analyse data from 212,102 vaccinated individuals within the REACT-2 programme in England, which uses self-administered lateral flow antibody tests in sequential cross-sectional community samples; 71,923 (33.9%) received at least one dose of BNT162b2 vaccine and 139,067 (65.6%) received ChAdOx1. For both vaccines, antibody positivity peaks 4-5 weeks after first dose and then declines. At least 21 days after second dose of BNT162b2, close to 100% of respondents test positive, while for ChAdOx1, this is significantly reduced, particularly in the oldest age groups (72.7% [70.9–74.4] at ages 75 years and above). For both vaccines, antibody positivity decreases with age, and is higher in females and those with previous infection. Antibody positivity is lower in transplant recipients, obese individuals, smokers and those with specific comorbidities. These groups will benefit from additional vaccine doses.

## Abstract (web summary)

The authors present results from the REACT-2 study, a series of cross-sectional community surveys during the SARS-CoV-2 pandemic in England. They measure antibodies by self-administered lateral flow tests and describe antibody positivity by time since vaccination, age, sex, co-morbidities, infection history, and vaccine type.

## Introduction

UK surveillance data of individuals testing positive for SARS-CoV-2 virus has shown COVID-19 vaccines to be highly effective against symptomatic infection and severe disease^1^. The pattern of antibody responses in the general population over time is less well characterized, particularly in relation to individual factors such as age, past infection and differing comorbidities.

Monitoring antibody prevalence at scale in population studies can provide insight into factors associated with vaccine immunogenicity and durability of responses. As part of the REal-Time Assessment for Community Transmission (REACT) study in England, over 900,000 individuals have performed self-testing lateral flow immunoassays (LFIAs) at home since mid-2020, making it one of the largest antibody testing surveillance programmes in the world. The results documented the extent of infection after the first wave^2,3^ and changes in the prevalence of antibody positivity over time due both to natural infection and vaccination^4^.

The UK vaccination programme began in December 2020 and by early September 2021 has delivered over 48 million first doses, predominantly using the ChAdOx1 (Astrazeneca, AZ) and BNT162b2 (Pfizer/BioNTech) vaccines. Here we report on the proportions of respondents testing positive (antibody positivity), and the estimated antibody prevalence in the population adjusted for LFIA performance, following at least one dose of vaccination by time since dose, assessed in rounds 5 (26 January–8 February 2021) and 6 (12–25 May 2021) of REACT-2.

## Results

There were 212,102 vaccinated individuals eligible for analysis; 71,923 (33.9%) self-reported receiving at least one dose of BNT162b2 vaccine, 139,067 (65.6%) ChAdOx1 vaccine, and 628 (0.3%) mRNA-1273 (Moderna); 484 (0.2%) did not know which vaccine they received.

**Fig. 1:**
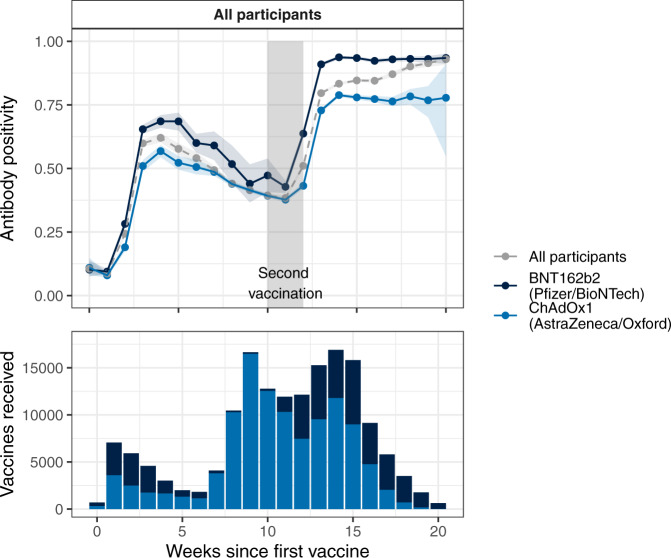
Vaccine response (upper panel) and uptake (lower panel) over time. Participants are grouped by weeks since first and second vaccination, and secondarily by vaccine received. The proportion of tests reported as positive (antibody positivity) within group by week is shown. Participants who had received either (i) a single dose but no second dose, or (ii) two doses of the vaccine between 10 and 12 weeks after the first were included in the plot. Shaded areas on either side of the plot lines denote 95% confidence intervals; the grey shaded block denotes the 10–12 week period after first vaccination. The upward trend post-second-dose observed in the all participants line is attributable to the changing proportions of ChAdOx1vs BNT162b2 vaccine after 17 weeks.

**Fig. 2:**
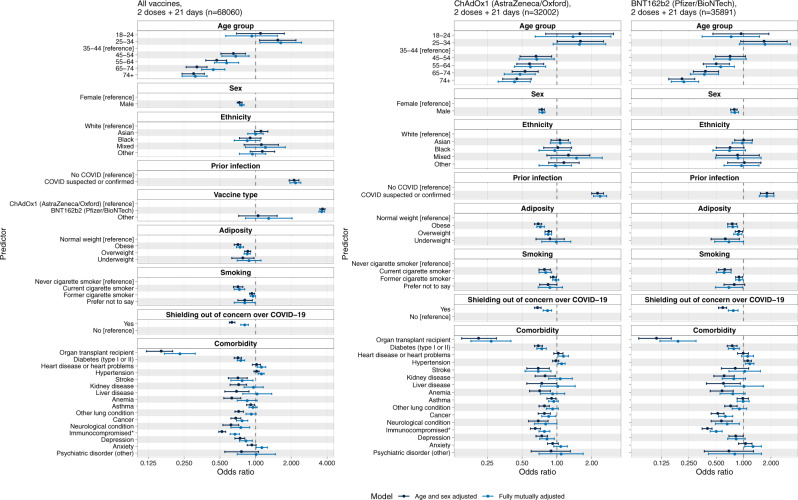
Forest plot showing odds ratios and 95% confidence intervals for antibody positivity (yes/no) versus biological and behavioural covariates in logistic regression models, among 68,060 respondents who had received two vaccine doses with the second dose at least 21 days prior. Dark blue CIs indicate models adjusted on age and sex only; light blue CIs indicate models adjusted on age, sex, ethnicity, adiposity, vaccine type (in the right panels only), prior infection, shielding status, comorbidities and smoking status.

Analysis is restricted to individuals who had their second dose between 10 and 12 weeks after the first, or who had their first vaccine <12 weeks earlier, as this was the standard regimen in the UK. Figure 1 shows the proportion of respondents who tested positive for antibodies on LFIA, by a number of weeks since their first or second vaccine dose. The proportion of individuals with detectable antibodies rose rapidly following first vaccination, peaking 4–5 weeks after initial dose. Antibody positivity fell gradually until the administration of the second dose (on average to 62.4% and 64.4% of the peak for BNT162b2 and ChAdOx1 respectively). It was lower in men than women at every timepoint (Fig. 2, supplementary Fig. 1), and decreased with age (supplementary Fig. 2). Antibody positivity was higher among those with a history of COVID-19 compared to those without, and in those receiving BNT162b2 compared to ChAdOx1 (Fig. 2; supplementary Fig. 3).

Following two doses of BNT162b2 vaccine, delivered on average 10.2 weeks apart, antibody positivity was >90% at all ages except 75 years and older, when it was slightly lower at 86.5% [85.5–87.5] (supplementary table 1). For ChAdOx1, antibody positivity after two doses, delivered on average 10.5 weeks apart, was high in younger age groups (for whom it is no longer recommended in the UK) but fell below 90% from 35 years upwards, declining to 72.7% [70.9–74.4] in the oldest age group. Positivity remained high following a second dose for up to 10 weeks for both vaccines overall, with a possible decline after 3–4 weeks in those aged 70+ years who had ChAdOx1 (supplementary Fig. S2).

A greater discrepancy in antibody positivity between age groups was observed following a single dose. After a single BNT162b2 dose, antibody positivity ranged from 91.5% [86.0–95.0] in those aged 18–29 to 37.6% [34.7–40.5] in those aged 70–79 years (supplementary table 1). For ChAdOx1 it was 64.9% [60.3–69.2] in the youngest age group and 25.0% [17.0–35.2] in the oldest age group (supplementary table 1). Of the small numbers who reported receiving mRNA-1273 vaccination, most were antibody positive after a single dose (supplementary table 3).

Multivariable regression analyses of 68,060 individuals who had received their second vaccine dose (either BNT162b2 or ChAdOx1) at least 21 days earlier showed that positivity was lower in those who were older with OR 0.30 [0.24, 0.37] for age 75+ vs reference category (35–44), higher in women than men (OR: 1.37 [1.30, 1.43]) and higher in individuals with prior suspected or confirmed COVID-19 than those with no such history (OR 2.39 [2.18–2.63]). Similar findings were observed for each vaccine separately (Fig. 2). Overall, antibody positivity was higher in those who received BNT162b2 rather than ChAdOx1 vaccine (OR 3.67 [3.49–3.85]) (Fig. 2).

Following two vaccine doses, antibody positivity was substantially lower (OR 0.16, 0.12–0.22) in people who reported being an organ transplant recipient or having a weakened immune system (as a result of either illness or treatment). Positivity was also lower in those with diabetes, stroke, kidney, liver, lung or neurological disease, cancer and depression (Fig. 2; supplementary table 4). Following a first dose of either vaccine type, there was higher antibody positivity in people of Black and Asian ethnicity than those of white ethnicity (supplementary Fig. 4), but this association was no longer apparent after the second dose.

## Discussion

The UK vaccination programme was atypical in initially recommending delay of second vaccination dose until 12 weeks after the first. In this context, small cohort studies have suggested a decline in antibody levels prior to second doses, although a delayed second dose may be associated with higher subsequent levels of antibodies^5^. Using two independent cross-sectional population samples, we confirmed a consistent pattern in antibody responses to vaccination, with antibody positivity peaking 3–4 weeks after first doses and then declining until after second doses were administered. A similar pattern was observed with both BNT162b2 and ChAdOx1 vaccines, though the initial peak was higher with the BNT162b2.

For both vaccines, there was a clear increase in the proportion of individuals testing positive after second doses. After two doses a very high proportion of individuals had detectable antibodies with little indication of subsequent waning although the maximum time since second dose was 10 weeks. The one exception was a trend towards lower antibody positivity 3–4 weeks after a second dose in those aged 70+ years who had ChAdOx1 (supplementary Fig. S2).

The implications of detectable IgG on LFIA testing are not yet well understood^6^. An absence of antibody does not necessarily imply vulnerability to infection, just as a positive result does not equate with protection. However, positivity on the LFIA used in this study was associated with the presence of neutralising antibody titres in a small cohort of healthcare workers^7^, and declining levels of neutralising antibody titres correlate with increased risk of symptomatic infection^8^, though this relationship is not clear for severe disease. Declining antibody positivity prior to second doses may be associated with increased vulnerability to infection, particularly in the setting of widespread circulation of Delta variant, which requires higher protective titres to achieve neutralisation^9^.

Evidence of declining antibody titres in older age groups and immunosuppressed individuals^10,11^ has been used to justify booster doses in these populations. However, correlates of protection remain undefined^6^, and antibody positivity is only one measure of a multifaceted immune response. SARS-CoV-2 vaccines have been shown to induce a polyfunctional Th1-dominated T cell response^12,13^, which persists for at least 6–8 months and continues to mature. B cell-mediated immunity can be sustained at least 12 months after initial infection^14^. It is possible many individuals will have sufficiently preserved immunity after two vaccine doses to prevent severe disease, but further follow up is required to determine the longevity of protection.

The findings of lower antibody positivity after ChAdOx1 vaccination, when compared with BNT162b2, are consistent with efficacy data from clinical trials^5,15^ and real-world findings^1,16^. However, within the UK vaccination programme different vaccines were prioritised for different groups over time and there should be caution in inferring superiority of one over another from studies of antibody responses alone. Of note, 33% of BNT162b2 recipients were healthcare workers, compared with just 5% of those who reported having ChAdOx1. Differences in antibody response persisted despite adjustment for age, sex, ethnicity, adiposity, prior infection, shielding, comorbidities and smoking status but there may be undetected differences between these groups. Head-to-head trials are ongoing and real world effectiveness data continue to emerge.

Large population studies such as this have the advantage of detecting small, but potentially biologically important, differences in vaccine immunogenicity. Across a range of vaccinations against other infections, increased age has been associated with reduced vaccine responses^17^. A similar effect has been seen in a number of SARS-CoV-2 vaccine cohorts^16,18–20^. We show a clear gradient of age response following two vaccination doses, with a greater difference following first doses.

Protective antibody responses are higher in females than males in response to a range of vaccines^17^. The mechanisms responsible are not well understood, but the fact that sex differences are preserved through advancing age groups suggests this is not solely explained by circulating levels of sex steroid hormones^17^. Early studies of the BNT162b2 vaccine have found similar disparity between the sexes^21^. Our data confirm higher antibody positivity amongst women for both BNT162b2 and ChAdOx1 vaccines. Unpicking these sex differences should be a priority for vaccine research and may further our grasp of sex differences in COVID-19 outcomes and Long-COVID.

A number of other host factors were associated with antibody positivity. Some, such as cancer and immunosuppression were expected. Immunosuppression had the largest effect on antibody positivity of any single factor, confirming, at a population level, findings from cohort studies^22^ which guided the decision to introduce third doses earlier to this group in the UK. Obesity was clearly associated with lower antibody response, as has been observed after COVID-19 mRNA vaccines^23^ and non-COVID-19 vaccines^17^. Given the poor outcomes from COVID-19 in obese individuals, this is a concerning result. Diabetes, stroke, chronic kidney disease, liver disease, neurological disease and depression were all associated with lower antibody positivity post-vaccination and remained so after we adjusted for time since vaccination. Heart disease and hypertension were not associated with lower antibody positivity.

There is a now well-recognised association between previous SARS-CoV-2 infection and higher antibody responses^16,24^, an effect confirmed in this study. Although the prior infection was accounted for when looking at the role of individual factors, we could not adjust for asymptomatic infections. The higher antibody positivity after a single vaccine dose in people of Black and Asian ethnicity has been described previously^16^. This may reflect a higher risk of natural infection that could prime subsequent vaccine responses and is not fully controlled for in our analysis which may exclude asymptomatic infection. Similarly, the association of individual comorbidities may be influenced by shielding behaviour. The association of smoking with lower antibody response is clear for both vaccines and, of note, has previously been documented after BNT162b2^23^.

Antibody surveillance using quantitative assays in centralised laboratories is challenging and expensive to deploy at scale, requiring significant effort in phlebotomy and sample transport. Self-administered lateral flow immunoassays (LFIAs) offer a well-validated qualitative approach that allows testing at a scale that can identify small, yet important, differences in population responses complementing more detailed cohort studies. The lower limit of detection for LFIAs is higher than laboratory assays and needs to be factored into analysis, particularly as lower levels of antibody may still represent important markers of protection against severe disease and/or hospitalisation. In addition, despite response rates of 26–28% across the two rounds, it is possible that the responders may not be representative of the population as a whole, limiting generalisability of our findings. Also, we relied on self-reported results on the LFIA which may introduce bias into the reporting of results. However, we did previously conduct a usability and acceptability study among a random sample of adults in the population which demonstrated high acceptability and usability of self-test LFIAs, including substantial concordance between participants and clinician interpreted results^25^. Despite these limitations, within-study comparisons, for example trends with age, should be relatively unaffected by such biases or test performance.

These population data confirm the importance of second vaccine doses and provide strong evidence for the role of individual factors (particularly age, sex, prior infection, adiposity and comorbidities) and vaccine type in determining antibody response, particularly after first doses. Whilst simplicity is key to successful vaccine roll-out, this information identifies key groups that may benefit from additional vaccine doses when available. Further data are needed to understand the heterogeneity and longevity of neutralising antibody response and cell-mediated immune responses, and the protection they offer against severe illness.

## Methods

REACT-2 methods have been published elsewhere^26,27^. Briefly, in each round of the study we invited a non-overlapping random community sample of adults aged 18 years or over in England, based on the National Health Service general practitioner registrations list. The sample was designed to provide approximately equal numbers of people in each of the 315 lower tier local authority (LTLA) areas in England. Informed consent was given via an online portal or via telephone. Those who registered were sent a Fortress lateral flow immunoassay (LFIA) test kit for SARS-CoV-2 antibody self-testing and asked to perform the test at home, report their test result and upload a photo of the completed test. In addition, they were asked to respond to a questionnaire that included details of COVID-19 vaccination type and date, self-reported comorbidities and any history of suspected or confirmed COVID-19 infection. Those who had not received a vaccine dose were asked whether they had been invited to take part in the vaccination programme and their response. For those who had not yet been offered a vaccine, we asked about their intention to accept. People who reported being unsure or who would decline vaccination were asked to select from a list of possible reasons for hesitancy^28^ with the option also of providing free-text responses.

For this analysis, participants were recruited in two successive rounds of sampling; round 5 (25 January to 8 February 2021) and round 6 **(**12 to 25 May 2021**)**. Round 6 adopted the design of previous rounds with the addition of a boosted sample of older adults to increase the power to detect whether the risk of becoming a case, being hospitalised or dying from COVID-19 differed between those testing positive and those testing negative on the LFIA following vaccination. Round 6 aimed for a total sample size of 240,000, with the inclusion of 70,000 additional people in age groups 55–64 and 65–67 years.

Round 5 included 155,172 adults who reported an IgG positive or negative result from their self-test; 26% of all those invited and 81% of those who registered submitted a test result. Round 6 included 207,337 adults who reported an IgG positive or negative result from their self-test; 28% of all those invited and 81% of those who registered submitted a test result.

The LFIA used in REACT-2 detects immune responses to the S1 subunit protein targeted by available vaccines. It was selected following evaluation of performance characteristics (sensitivity and specificity) against pre-defined criteria for detection of IgG^29^, with extensive public involvement and user testing^25^. We estimated clinical sensitivity of the LFIA on finger-prick blood (self-read) for IgG antibodies following natural infection at 84.4% (70.5, 93.5) among RT-PCR confirmed cases in healthcare workers and specificity 98.6% (97.1, 99.4) in pre-pandemic sera^29,30^. In a further study in over 5000 non-healthcare key workers using the same kit and instructions, we showed that self-test LFIAs had a sensitivity of 82.1% (95% CI, 77.7–86.0) and specificity of 97.8% (95% CI, 97.3–98.2) compared with Abbott ELISA^31^. We also found a high concordance of self-reported clinician-read results from the uploaded photographs^25^.

Positivity was calculated as the proportion of individuals with a positive IgG result on the LFIA of all those with a valid (IgG positive or negative) result. For analyses at population level (but not for individual vaccine response) we adjusted for test performance^7,32^. We used multivariable logistic regression to estimate odds of antibody positivity adjusting for potential confounders. These included age, sex, days since vaccine and self-reported previous COVID-19 infection status, as well as interaction effects between age and sex.

Data were analysed using the statistical package R version 4.0.0. We obtained research ethics approval from the South Central-Berkshire B Research Ethics Committee (IRAS ID: 283787), and Medicines and Healthcare products Regulatory Agency approval for use of the LFIA for research purposes only.

### Public involvement

The REACT Public Advisory Panel has provided regular review and revision of the study processes and results.

### Reporting summary

Further information on research design is available in the Nature Research Reporting Summary linked to this article.

## Supplementary information

Supplementary Information

Reporting Summary

## Data Availability

The data generated in this study have been deposited in a GitHub database under an accession code available here. The data are available under unrestricted access. Requests for access to raw data should be addressed to the corresponding authors and will be answered within 12 weeks. The raw data are protected and are not available due to data privacy laws. The processed data are available at GitHub. All data generated in this study are provided in the Supplementary Information/Source Data file.
