# Supplementary material for: Population antibody responses following COVID-19 vaccination in 212,102 individuals
Source: Nat Commun. 2022 Feb 16;13:907. doi: 10.1038/s41467-022-28527-x (PMC8850615; doi:10.1038/s41467-022-28527-x)
Supplement: Supplementary file 1 — Supplementary Information [file 41467_2022_28527_MOESM1_ESM.pdf]

Supplementary table 1
Antibody positivity by age group, unadjusted and adjusted for known test performance, stratified by vaccine type received and number of doses.

A. ChAdOx1 (AstraZeneca)

ChAdOx1 (AstraZeneca): one dose				
**Category**	Positive	Total	Antibody (Ab) positivity	Ab positivity adjusted for test performance
**18-24**	283	436	64.9 [60.3-69.2]	68.4 [63.0-73.4]
**25-34**	798	1338	59.6 [57.0-62.2]	62.2 [59.1-65.3]
**35-44**	1572	2962	53.1 [51.3-54.9]	54.5 [52.4-56.6]
**45-54**	4606	10006	46.0 [45.1-47.0]	46.3 [45.2-47.4]
**55-64**	16608	41907	39.6 [39.2-40.1]	38.8 [38.3-39.4]
**65-74**	1115	3115	35.8 [34.1-37.5]	34.3 [32.4-36.3]
**75+**	21	84	25.0 [17.0-35.2]	21.7 [12.3-33.6]
**Two doses**				
**18-24**	110	121	90.9 [84.5-94.8]	98.8 [91.2-100.0]
**25-34**	298	328	90.9 [87.2-93.5]	98.7 [94.5-100.0]
**35-44**	410	475	86.3 [82.9-89.1]	93.4 [89.5-96.7]
**45-54**	658	820	80.2 [77.4-82.8]	86.3 [83.0-89.3]
**55-64**	4446	5703	78.0 [76.9-79.0]	83.6 [82.4-84.9]
**65-74**	15975	21082	75.8 [75.2-76.3]	81.1 [80.4-81.8]
**75+**	1767	2431	72.7 [70.9-74.4]	77.5 [75.4-79.5]

B. BNT162b2 (Pfizer)

BNT162b2 (Pfizer): one dose				
**Category**	Positive	Total	Antibody (Ab) positivity	Ab positivity adjusted for test performance
**18-24**	140	153	91.5 [86.0-95.0]	99.5 [93.1-100.0]
**25-34**	370	426	86.9 [83.3-89.7]	94.1 [89.9-97.4]
**35-44**	475	593	80.1 [76.7-83.1]	86.2 [82.2-89.7]
**45-54**	601	893	67.3 [64.2-70.3]	71.2 [67.5-74.7]
**55-64**	935	1865	50.1 [47.9-52.4]	51.1 [48.4-53.7]
**65-74**	226	526	43.0 [38.8-47.2]	42.7 [37.8-47.7]
**75+**	399	1062	37.6 [34.7-40.5]	36.4 [33.1-39.9]
**Two doses**				
**18-24**	307	317	96.8 [94.3-98.3]	100.0 [100.0-100.0]
**25-34**	864	880	98.2 [97.1-98.9]	100.0 [100.0-100.0]
**35-44**	1293	1333	97.0 [95.9-97.8]	100.0 [100.0-100.0]
**45-54**	1880	1962	95.8 [94.8-96.6]	100.0 [100.0-100.0]
**55-64**	9001	9567	94.1 [93.6-94.5]	100.0 [100.0-100.0]
**65-74**	14782	16058	92.1 [91.6-92.5]	100.0 [99.6-100.0]
**75+**	4067	4701	86.5 [85.5-87.5]	93.7 [92.5-94.8]

Supplementary table 2
Characteristics of study population, by reported vaccination type

Variable	Category	ChAdOx1 (AstraZeneca)	BNT162b2 (Pfizer)	Moderna	Don't know	Sum / mean (SD)	Weighted proportions / means (SDs) from full REACT-2 cohort (n=953,088)*
**All participants**	All participants	139,067 (100%)	71,923 (100%)	628 (100%)	484 (100%)	212,102	100%
**Age**	Mean (SD)	60.96 (10.11)	62.88 (13.22)	45.97 (6.83)	65.03 (15.8)	61.58 (11.34)	52.98 (15.99)
**Sex**	Female	76,361 (54.9%)	42,791 (59.5%)	339 (54%)	247 (51%)	119,738	56.10%
	Male	62,705 (45.1%)	29,131 (40.5%)	289 (46%)	237 (49%)	92,362	43.90%
**Ethnicity**	Asian	3,308 (2.4%)	2,400 (3.4%)	30 (4.8%)	23 (4.8%)	5,761	3.50%
	Black	1,267 (0.9%)	714 (1%)	9 (1.4%)	7 (1.5%)	1,997	0.90%
	Mixed	971 (0.7%)	493 (0.7%)	10 (1.6%)	3 (0.6%)	1,477	1.20%
	Other	1,041 (0.8%)	658 (0.9%)	8 (1.3%)	6 (1.3%)	1,713	0.90%
	White	131,494 (95.2%)	67,147 (94%)	570 (90.9%)	436 (91.8%)	199,647	93.60%
**Healthcare worker**	No	65,144 (94.7%)	20,226 (62.6%)	549 (98.4%)	147 (85.5%)	86,066	92%
	Yes	3,610 (5.3%)	12,103 (37.4%)	9 (1.6%)	25 (14.5%)	15,747	8%
**Number of comorbidities**	None	79,211 (57%)	34,436 (47.9%)	475 (75.6%)	225 (46.5%)	114,347	58.10%
	1	37,923 (27.3%)	22,482 (31.3%)	108 (17.2%)	123 (25.4%)	60,636	26%
	2	14,454 (10.4%)	9,749 (13.6%)	32 (5.1%)	76 (15.7%)	24,311	10.60%
	3+	7,479 (5.4%)	5,256 (7.3%)	13 (2.1%)	60 (12.4%)	12,808	5.30%

*Samples are weighted to be representative of the adult England population in terms of age, sex, ethnicity, region and deprivation. Within-category proportions (of categorical variables) and means (of continuous variables) should therefore be representative of the adult England population.

Supplementary table 3
Antibody prevalence within study population, by prior infection status.

		Prior COVID infection (suspected or confirmed)				No prior COVID infection			
**Variable**	**Category**	**LFT Positive**	**Total**	**Antibody positivity**	**Prevalence adjusted for test performance**	**LFT Positive**	**Total**	**Antibody positivity**	**Prevalence adjusted for test performance**
**All participants**	All participants	20165	25428	79.3 [78.8-79.8]	85.2 [84.6-85.8]	109965	180333	61.0 [60.8-61.2]	63.8 [63.5-64.0]
**Age group (years)**	18-24	356	404	88.1 [84.6-90.9]	95.5 [91.4-98.8]	910	1297	70.2 [67.6-72.6]	74.5 [71.5-77.4]
	25-34	934	1114	83.8 [81.6-85.9]	90.5 [87.9-92.9]	2550	3753	67.9 [66.4-69.4]	71.9 [70.2-73.7]
	35-44	1520	2080	73.1 [71.1-74.9]	77.9 [75.7-80.1]	4625	8618	53.7 [52.6-54.7]	55.2 [54.0-56.5]
	45-54	2616	3397	77.0 [75.6-78.4]	82.5 [80.8-84.2]	8221	15635	52.6 [51.8-53.4]	54.0 [53.0-54.9]
	55-64	8988	11524	78.0 [77.2-78.7]	83.7 [82.8-84.6]	36791	69990	52.6 [52.2-52.9]	53.9 [53.5-54.4]
	65-74	5186	6069	85.5 [84.5-86.3]	92.4 [91.3-93.4]	49988	67784	73.7 [73.4-74.1]	78.7 [78.3-79.1]
	74+	565	840	67.3 [64.0-70.4]	71.1 [67.3-74.7]	6880	13256	51.9 [51.1-52.8]	53.2 [52.2-54.2]
**Sex**	Female	12156	15011	81.0 [80.3-81.6]	87.2 [86.4-87.9]	64762	101227	64.0 [63.7-64.3]	67.3 [66.9-67.6]
	Male	8008	10416	76.9 [76.1-77.7]	82.4 [81.4-83.3]	45202	79105	57.1 [56.8-57.5]	59.3 [58.9-59.7]
**Ethnicity**	Asian	783	889	88.1 [85.8-90.0]	95.5 [92.8-97.8]	3272	4707	69.5 [68.2-70.8]	73.8 [72.2-75.3]
	Black	288	333	86.5 [82.4-89.7]	93.6 [88.8-97.4]	1077	1597	67.4 [65.1-69.7]	71.3 [68.6-74.0]
	Mixed	182	235	77.4 [71.7-82.3]	83.0 [76.3-88.8]	764	1190	64.2 [61.4-66.9]	67.6 [64.3-70.7]
	Other	229	263	87.1 [82.5-90.6]	94.3 [88.9-98.4]	925	1387	66.7 [64.2-69.1]	70.5 [67.5-73.3]
	White	18529	23504	78.8 [78.3-79.4]	84.7 [84.1-85.3]	103193	170208	60.6 [60.4-60.9]	63.4 [63.1-63.6]
**Adioposity**	Normal weight	6227	7799	79.8 [78.9-80.7]	85.9 [84.8-86.9]	39141	61825	63.3 [62.9-63.7]	66.5 [66.1-67.0]
	Obese	4584	5821	78.7 [77.7-79.8]	84.6 [83.3-85.8]	20810	35121	59.3 [58.7-59.8]	61.8 [61.2-62.4]
	Overweight	6934	8685	79.8 [79.0-80.7]	85.8 [84.8-86.8]	36517	60579	60.3 [59.9-60.7]	63.0 [62.5-63.4]
	Underweight	150	193	77.7 [71.3-83.0]	83.4 [75.9-89.6]	1095	1722	63.6 [61.3-65.8]	66.8 [64.1-69.5]
**Smoking**	Current cigarette smoker	1184	1708	69.3 [67.1-71.5]	73.5 [70.9-76.0]	6473	11987	54.0 [53.1-54.9]	55.6 [54.6-56.7]

	Former cigarette smoker	7085	8944	79.2 [78.4-80.0]	85.1 [84.1-86.1]	37716	61527	61.3 [60.9-61.7]	64.2 [63.7-64.6]
	Never cigarette smoker	11349	14041	80.8 [80.2-81.5]	87.0 [86.2-87.8]	63154	102518	61.6 [61.3-61.9]	64.5 [64.2-64.9]
	Prefer not to say	483	628	76.9 [73.5-80.0]	82.4 [78.4-86.1]	2231	3662	60.9 [59.3-62.5]	63.7 [61.9-65.6]
**Vaccine type**	ChAdOx1 (AstraZeneca)	12695	16860	75.3 [74.6-75.9]	80.5 [79.8-81.3]	62050	117985	52.6 [52.3-52.9]	54.0 [53.6-54.3]
	Don't know	35	51	68.6 [55.0-79.7]	72.7 [56.8-85.7]	167	408	40.9 [36.3-45.8]	40.3 [34.9-46.0]
	mRNA-1273 (Moderna)	81	98	82.7 [74.0-88.9]	89.1 [79.0-96.4]	392	516	76.0 [72.1-79.5]	81.3 [76.8-85.4]
	BNT162b2 (Pfizer)	7343	8407	87.3 [86.6-88.0]	94.6 [93.8-95.4]	47308	61362	77.1 [76.8-77.4]	82.6 [82.2-83.0]
**Number of vaccine doses reported**	One dose	8704	12640	68.9 [68.0-69.7]	73.0 [72.1-73.9]	24461	71285	34.3 [34.0-34.7]	32.6 [32.2-33.0]
	Two doses	11456	12783	89.6 [89.1-90.1]	97.3 [96.7-97.9]	85490	109028	78.4 [78.2-78.7]	84.2 [83.9-84.5]
	Unvaccinated	5	5	100.0 [64.9-100.0]	100.0 [68.4-100.0]	14	20	70.0 [48.1-85.5]	74.3 [48.7-92.4]
**Shielding out of concern over COVID-19**	No	18714	23378	80.0 [79.5-80.6]	86.1 [85.5-86.7]	98112	159575	61.5 [61.2-61.7]	64.4 [64.1-64.7]
	Yes	1451	2050	70.8 [68.8-72.7]	75.2 [72.9-77.5]	11853	20758	57.1 [56.4-57.8]	59.2 [58.5-60.0]
**Comorbiditi es**	Organ transplant recipient	21	30	70.0 [52.1-83.3]	74.3 [53.4-89.9]	128	319	40.1 [34.9-45.6]	39.4 [33.3-45.8]
	Diabetes (type I or II)	1475	1837	80.3 [78.4-82.0]	86.4 [84.2-88.4]	8938	14318	62.4 [61.6-63.2]	65.5 [64.5-66.4]
	Heart disease or heart problems	1139	1419	80.3 [78.1-82.3]	86.3 [83.8-88.7]	7277	11053	65.8 [64.9-66.7]	69.5 [68.4-70.5]
	Hypertension (high blood pressure)	3654	4547	80.4 [79.2-81.5]	86.5 [85.1-87.8]	22549	35405	63.7 [63.2-64.2]	67.0 [66.4-67.5]
	Stroke	218	268	81.3 [76.2-85.6]	87.6 [81.6-92.5]	1219	1903	64.1 [61.9-66.2]	67.4 [64.8-69.9]

	Kidney disease	137	170	80.6 [74.0-85.8]	86.7 [79.0-92.9]	632	1097	57.6 [54.7-60.5]	59.8 [56.4-63.2]
	Liver disease	268	338	79.3 [74.7-83.3]	85.2 [79.8-89.9]	1209	1942	62.3 [60.1-64.4]	65.3 [62.7-67.8]
	Anemia	2150	2743	78.4 [76.8-79.9]	84.1 [82.3-85.9]	9749	15049	64.8 [64.0-65.5]	68.2 [67.3-69.1]
	Asthma	781	1004	77.8 [75.1-80.3]	83.4 [80.3-86.3]	4521	7042	64.2 [63.1-65.3]	67.6 [66.2-68.9]
	Other lung condition	378	485	77.9 [74.0-81.4]	83.6 [79.1-87.7]	2956	4722	62.6 [61.2-64.0]	65.7 [64.1-67.3]
	Cancer	219	301	72.8 [67.5-77.5]	77.6 [71.4-83.1]	1457	2683	54.3 [52.4-56.2]	56.0 [53.8-58.2]
	Condition affecting the brain and nerves	831	1043	79.7 [77.1-82.0]	85.7 [82.7-88.4]	5037	7657	65.8 [64.7-66.8]	69.4 [68.2-70.6]
	A weakened immune system/reduced ability to deal with infections	1438	1898	75.8 [73.8-77.6]	81.1 [78.8-83.3]	5659	10075	56.2 [55.2-57.1]	58.2 [57.0-59.3]
	Depression	2051	2668	76.9 [75.2-78.4]	82.4 [80.5-84.2]	8054	13386	60.2 [59.3-61.0]	62.8 [61.9-63.8]
	Anxiety	301	428	70.3 [65.8-74.5]	74.7 [69.5-79.6]	822	1907	43.1 [40.9-45.3]	42.9 [40.3-45.5]
	Psychiatric disorder	9854	12267	80.3 [79.6-81.0]	86.4 [85.6-87.2]	55423	90041	61.6 [61.2-61.9]	64.5 [64.1-64.8]

Supplementary table 4
Odds ratios (95% CIs) for antibody positivity among 68,060 double vaccinated (plus 21 days) participants, for key covariates

Variable	Category	Adjusted on age and sex	Mutually adjusted: on age, sex, ethnicity, previous COVID-19 infection, vaccination status, adiposity, smoking status, shielding status and all comorbidities
**Age group (years)**	35-44 (reference)	-	-
	18-24	1.1 (0.69,1.75)	0.93 (0.56,1.53)
	25-34	1.54 (1.08,2.19)	1.63 (1.09,2.44)
	45-54	0.65 (0.51,0.82)	0.68 (0.52,0.88)
	55-64	0.47 (0.38,0.57)	0.57 (0.45,0.72)
	65-74	0.32 (0.26,0.39)	0.44 (0.35,0.55)
	74+	0.3 (0.24,0.37)	0.31 (0.24,0.39)
**Sex**	Female (reference)	-	-
	Male	0.73 (0.7,0.77)	0.76 (0.73,0.8)
**Ethnicity**	White (reference)	-	-
	Asian	1.11 (0.98,1.27)	1 (0.86,1.16)
	Black	0.9 (0.73,1.11)	0.85 (0.66,1.09)
	Mixed	1.12 (0.8,1.56)	1.21 (0.82,1.78)
	Other	1.14 (0.9,1.45)	0.94 (0.73,1.22)
**Prior infection**	No COVID-19 (reference)	-	-
	COVID-19 suspected or confirmed	2.11 (1.92,2.32)	2.17 (1.95,2.41)
**Vaccine type**	ChAdOx1 (AstraZeneca)	-	-
	BNT162b2 (Pfizer)	1.43 (0.41,4.92)	3.61 (3.42,3.81)
	Other	3.67 (3.49,3.85)	1.29 (0.82,2.03)
**Adiposity**	Normal weight (reference)	-	-
	Obese	0.71 (0.66,0.75)	0.74 (0.69,0.79)
	Overweight	0.86 (0.81,0.91)	0.85 (0.8,0.9)
	Underweight	0.78 (0.63,0.97)	0.88 (0.7,1.11)
**Smoking**	Never cigarette smoker (reference)	-	-
	Current cigarette smoker	0.71 (0.65,0.78)	0.73 (0.66,0.81)
	Former cigarette smoker	0.93 (0.89,0.98)	0.95 (0.9,1)
	Prefer not to say	0.81 (0.71,0.94)	0.81 (0.66,0.99)
**Shielding out of concern over COVID-19**	No (reference)	-	-
	Yes	0.63 (0.6,0.67)	0.81 (0.75,0.87)
**Comorbidities**	Organ transplant recipient	0.16 (0.12,0.2)	0.23 (0.17,0.31)
	Diabetes (type I or II)	0.71 (0.66,0.76)	0.75 (0.7,0.81)
	Heart disease or heart problems	1.02 (0.94,1.1)	1.12 (1.03,1.22)

	Hypertension	1.02 (0.97,1.07)	1.12 (1.05,1.18)
	Stroke	0.71 (0.59,0.85)	0.77 (0.62,0.95)
	Kidney disease	0.72 (0.61,0.84)	0.96 (0.8,1.16)
	Liver disease	0.69 (0.55,0.88)	1.03 (0.78,1.37)
	Anemia	0.63 (0.54,0.75)	0.85 (0.7,1.03)
	Asthma	0.91 (0.84,0.98)	0.95 (0.88,1.03)
	Other lung condition	0.72 (0.67,0.79)	0.92 (0.83,1.01)
	Cancer	0.68 (0.62,0.75)	0.77 (0.69,0.86)
	Neurological condition	0.62 (0.53,0.72)	0.75 (0.62,0.89)
	Immunocompromised*	0.52 (0.49,0.56)	0.67 (0.61,0.73)
	Depression	0.74 (0.67,0.81)	0.83 (0.73,0.94)
	Anxiety	0.93 (0.85,1.01)	1.13 (1.01,1.26)
	Psychiatric disorder (other)	0.76 (0.55,1.06)	1.01 (0.7,1.47)

Supplementary figure 1

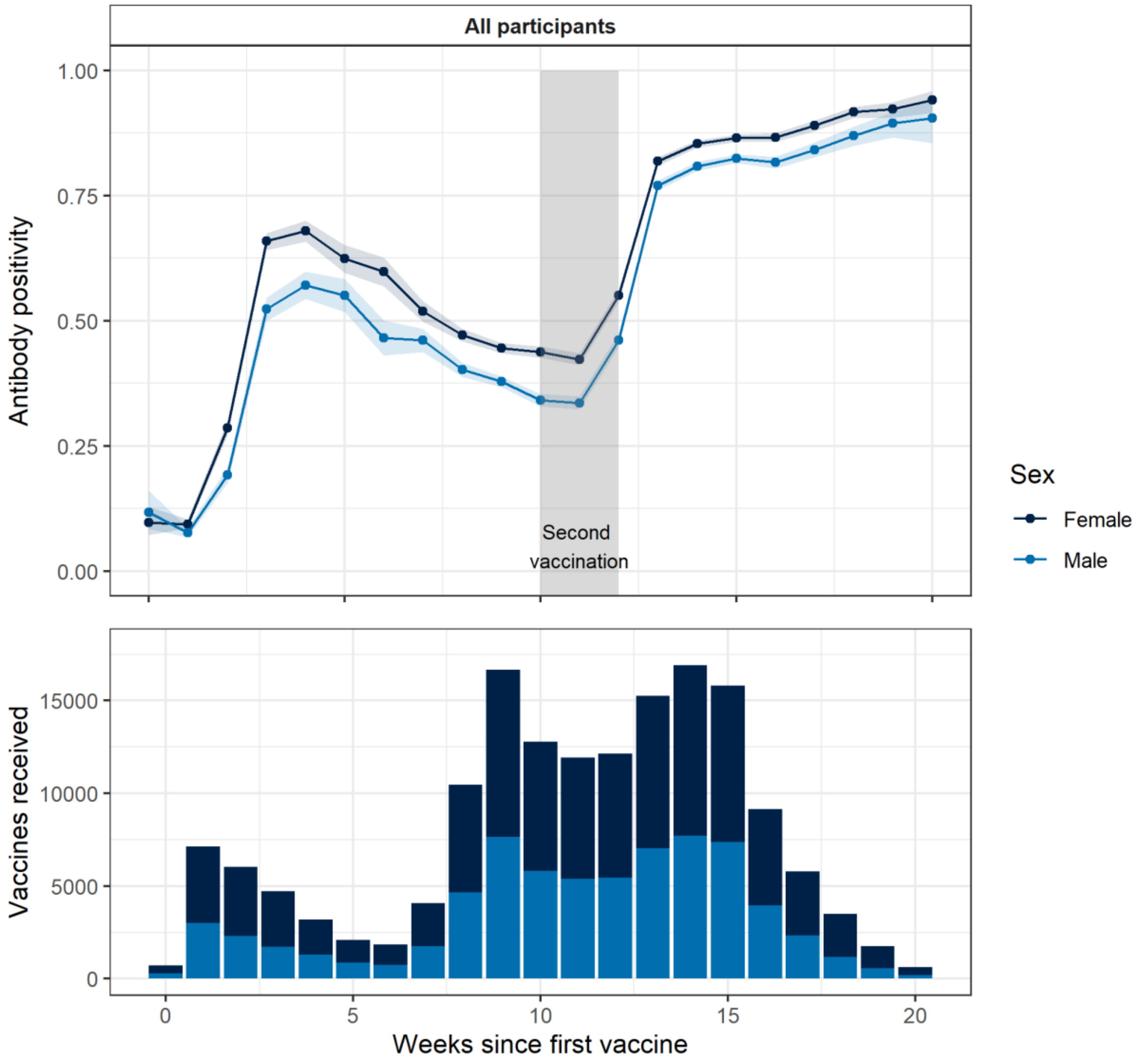

Supplementary figure 1 Vaccine response (upper panel) and uptake (lower panel) over time, by sex. Participants are grouped by weeks since first and second vaccination, and secondarily by vaccine reported. The proportion of tests reported as positive (antibody positivity) within group by week is shown. Participants who had received either (i) a single dose but no second dose, or (ii) two doses of the vaccine between 10 and 12 weeks after the first were included in the plot. Shaded areas denote 95% confidence intervals. The upward trend post-second-dose observed is attributable to the changing proportions of AZ vs Pfizer vaccine after 17 weeks.

Supplementary figure 2

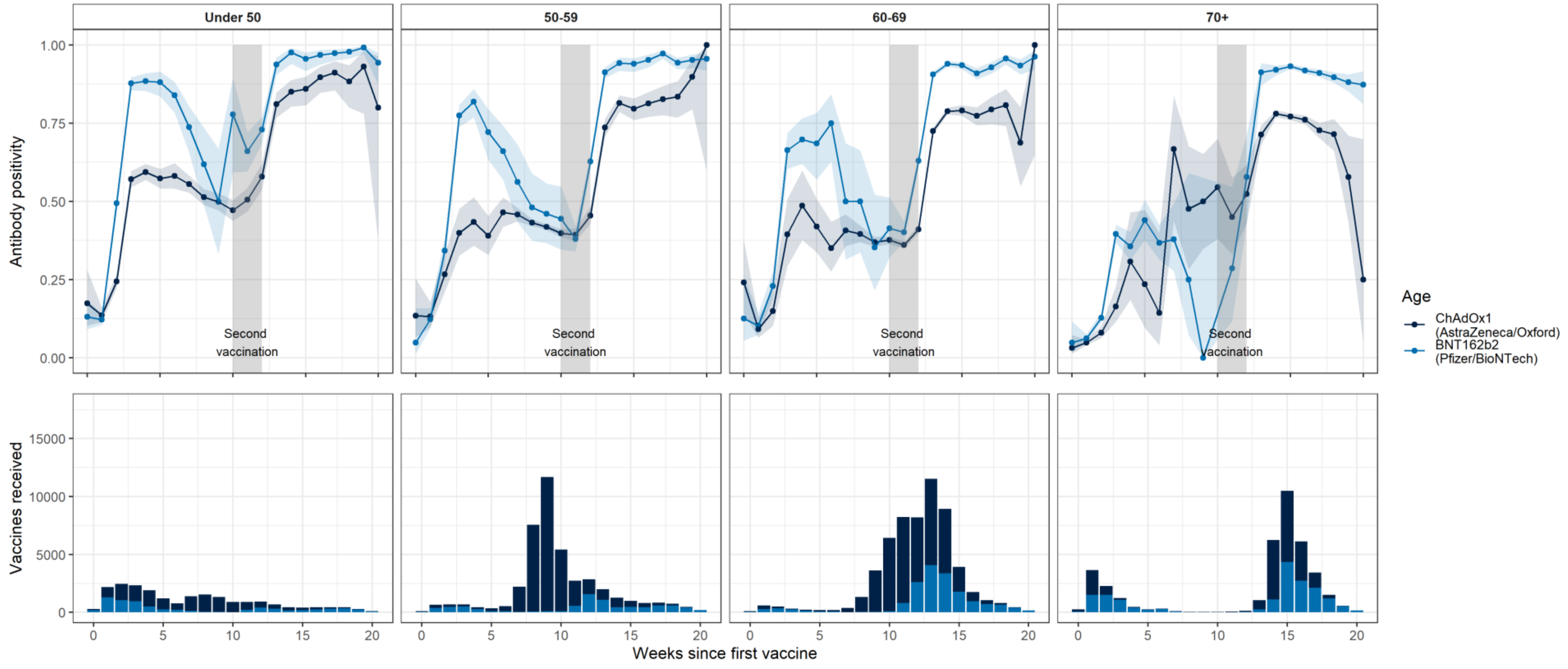

Supplementary figure 2 Vaccine response (upper panel) and uptake (lower panel) over time, by age. Figure specifications are as in Supplementary figure 1.

Supplementary figure 3

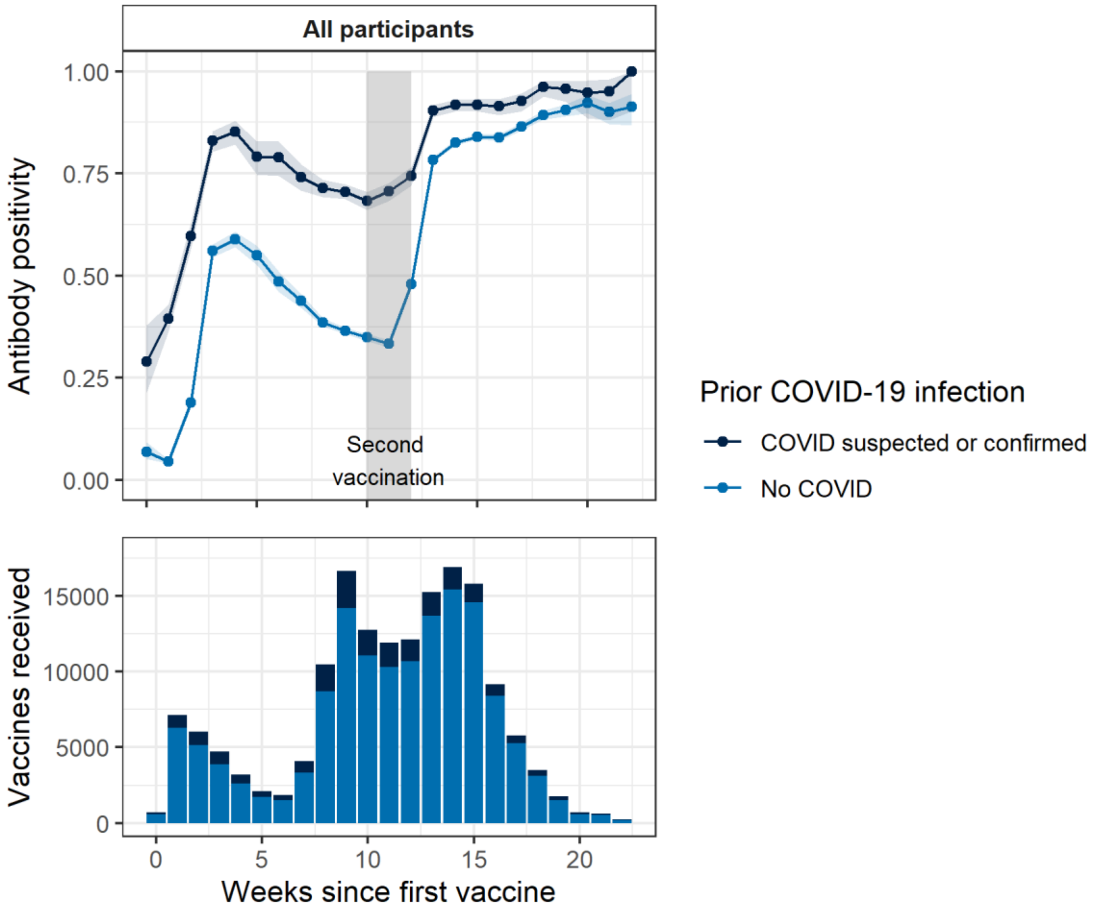

Supplementary figure 3 Vaccine response (upper panel) and uptake (lower panel) over time, by prior infection status. Figure specifications are as in Supplementary Figure 1.

Supplementary figure 4

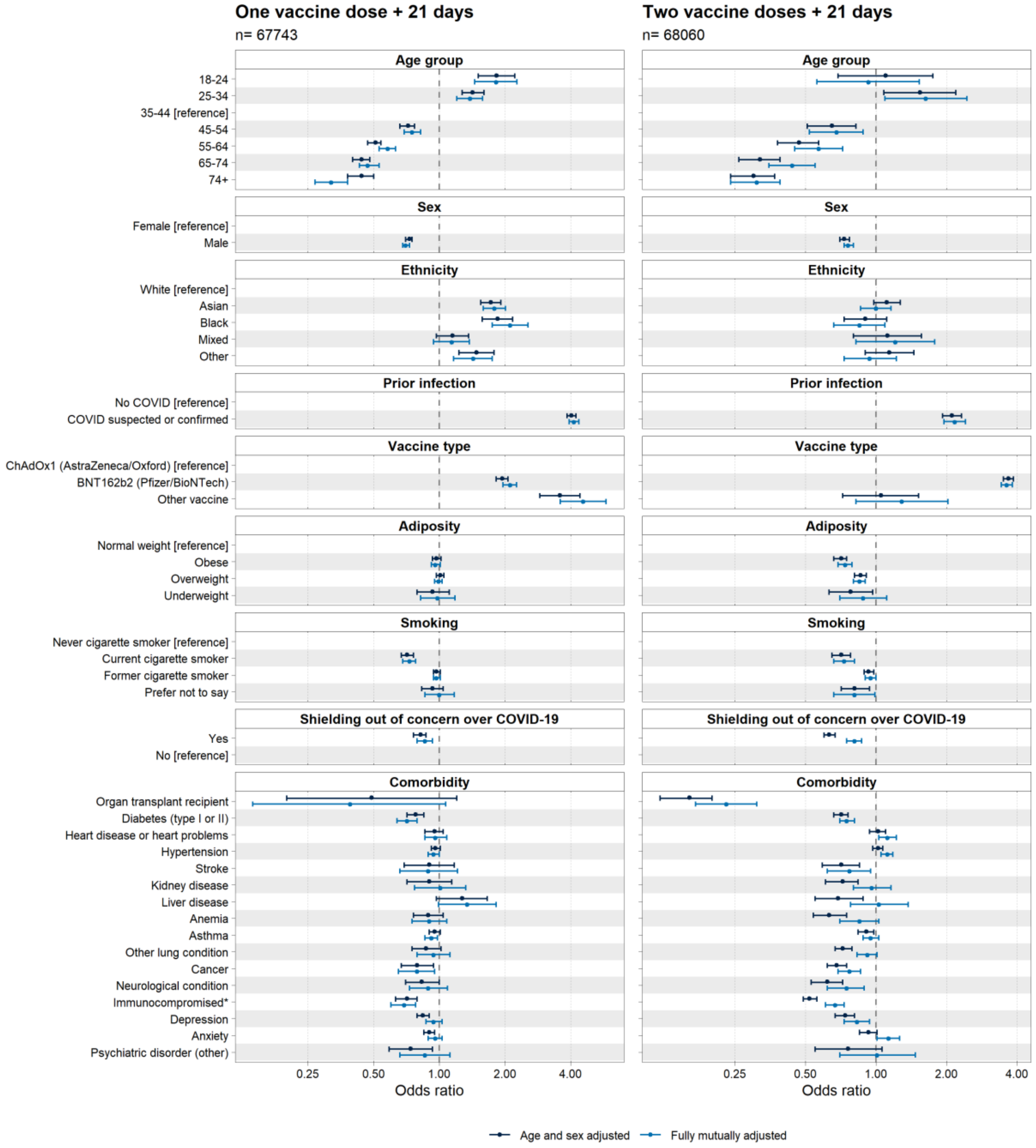

Supplementary figure 4: Forest plot showing odds ratios and 95% confidence intervals for antibody positivity (yes/no) versus biological and behavioural covariates in logistic models, among 135,803 respondents who had received (i) one vaccine at least 21 days ago (left panel, n=67,743), and (ii) two vaccine doses with the second dose at least 21 days prior (right panel, n=68,060). Dark blue CIs indicate models adjusted on age and sex only; light blue CIs indicated models adjusted on age, sex, ethnicity, adiposity, vaccine type, prior infection, shielding status, comorbidities and smoking status.

Supplementary figure 5

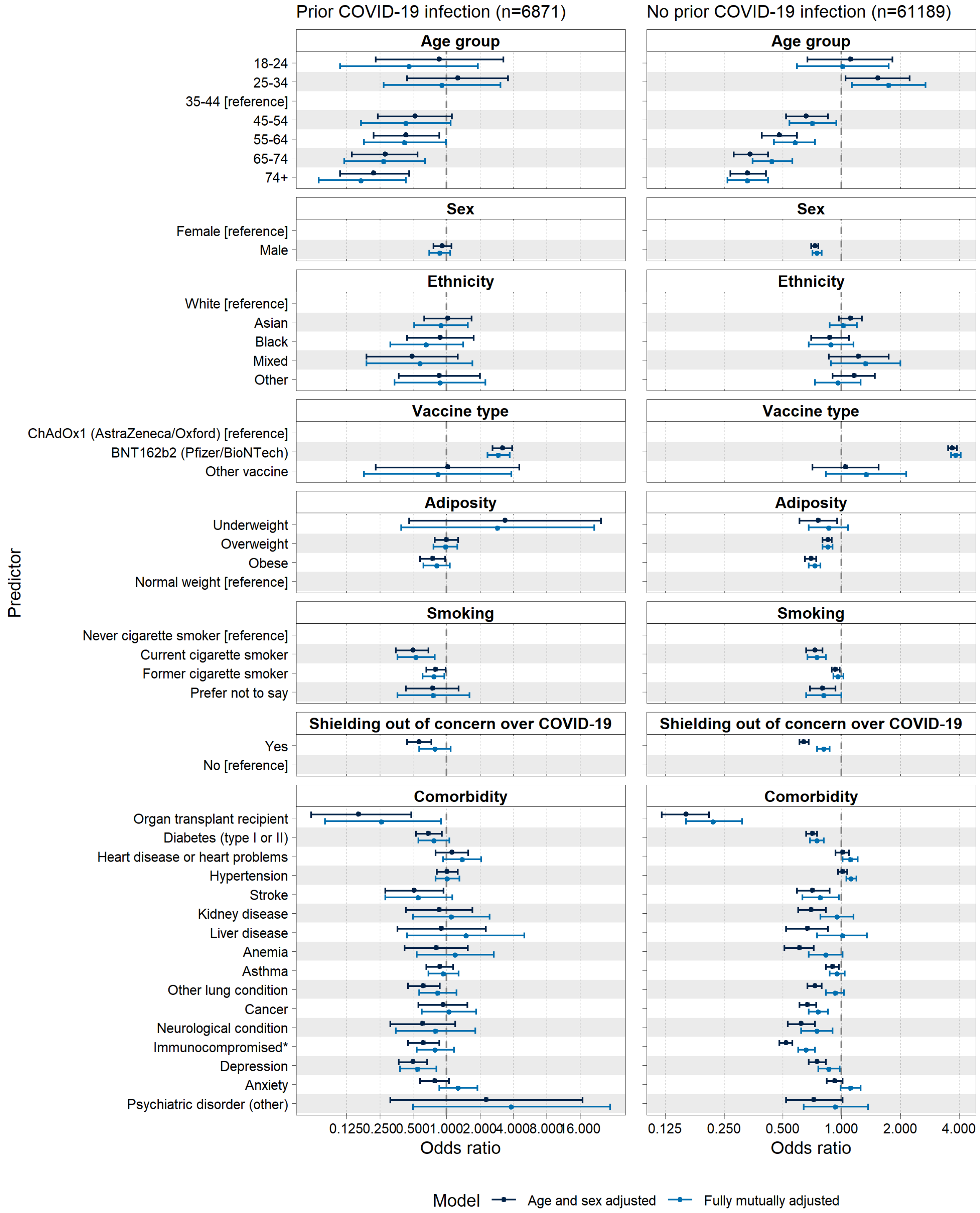

Supplementary figure 5: Forest plot showing odds ratios among 68,060 double-vaccinated (+21 days) respondents who reported prior COVID-19 infection (left panel, n=6,871), or no prior COVID-19 infection (right panel, n=61,189). Dark blue CIs indicate models adjusted on age and sex only; light blue CIs indicated models adjusted on age, sex, ethnicity, adiposity, vaccine type, shielding status, comorbidities and smoking status.

.

Supplementary figure 6

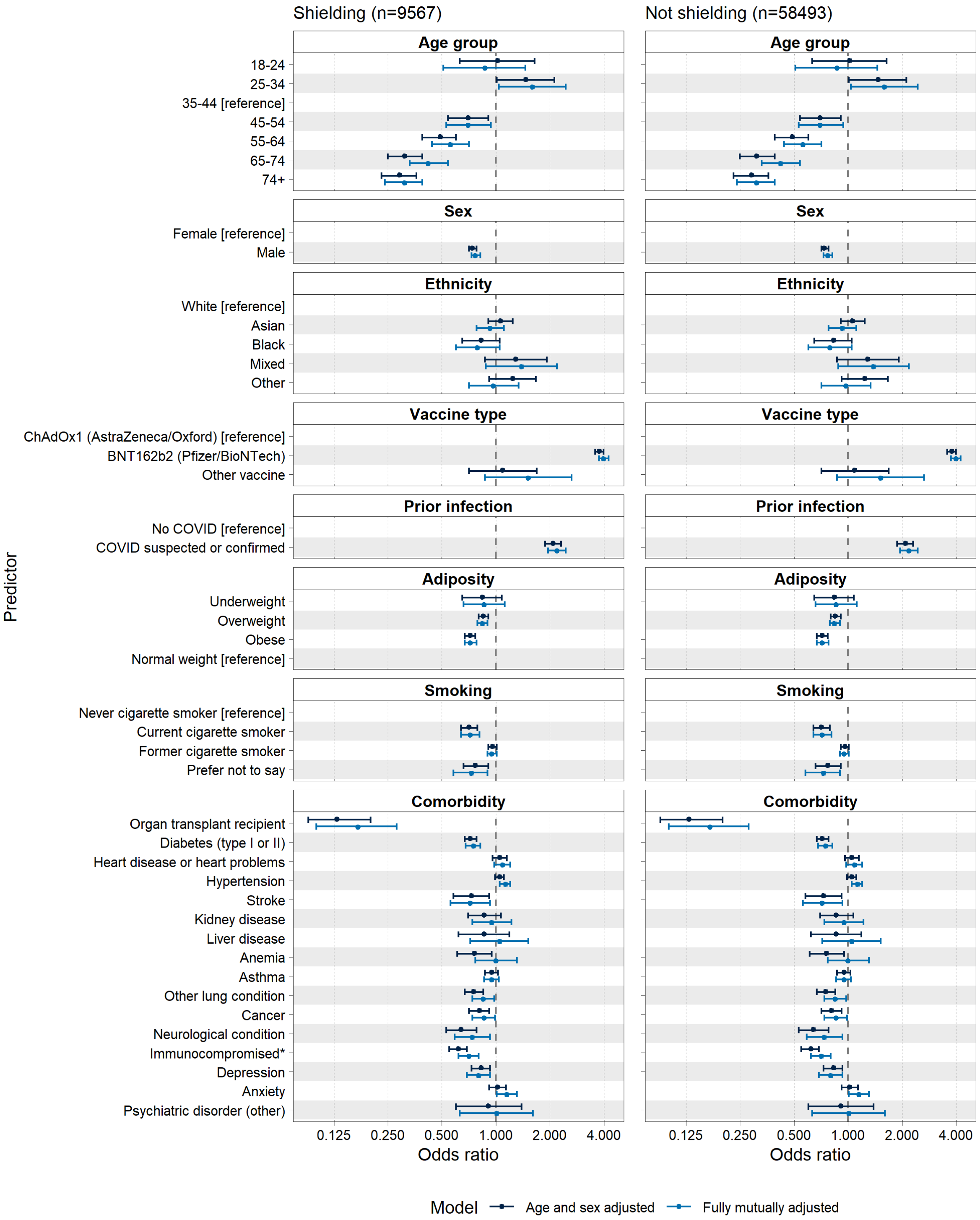

Supplementary figure 6 Forest plot showing odds ratios among 68,060 double-vaccinated (+21 days) respondents who were shielding (left panel, n=9,567), or not shielding (right panel, n=58,493). Dark blue CIs indicate models adjusted on age and sex only; light blue CIs indicated models adjusted on age, sex, ethnicity, adiposity, vaccine type, past infection, comorbidities and smoking status.

Supplementary figure 7

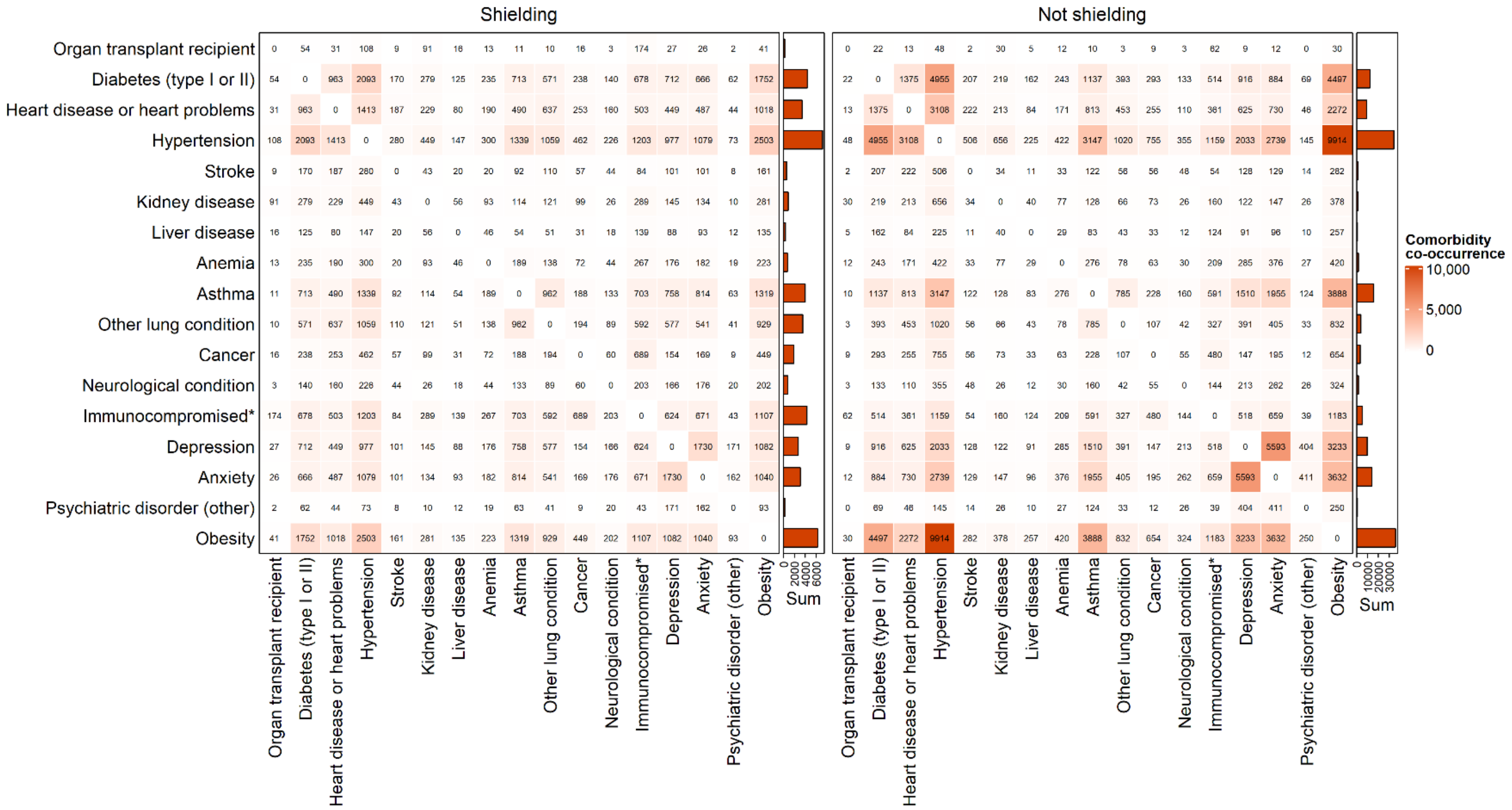

Supplementary figure 7 Heatmap showing co-occurrence of comorbidities in participants who reported that they were shielding (left panel) or not shielding (right panel)

END OF DOCUMENT
