# Supplementary material for: Population antibody responses following COVID-19 vaccination in 212,102 individuals
Source: Nat Commun. 2022 Feb 16;13:907. doi: 10.1038/s41467-022-28527-x (PMC8850615; doi:10.1038/s41467-022-28527-x)
Supplement: Supplementary file 2 — Reporting Summary [file 41467_2022_28527_MOESM2_ESM.pdf]

nature portfolio

Corresponding author(s): Graham Cooke
Helen Ward

Last updated by author(s): Dec 7, 2021

Reporting Summary

Nature Portfolio wishes to improve the reproducibility of the work that we publish. This form provides structure for consistency and transparency in reporting. For further information on Nature Portfolio policies, see our Editorial Policies and the Editorial Policy Checklist.

Statistics

For all statistical analyses, confirm that the following items are present in the figure legend, table legend, main text, or Methods section.

n/a	Confirmed	
☐	☒	The exact sample size (*n*) for each experimental group/condition, given as a discrete number and unit of measurement
☒	☐	A statement on whether measurements were taken from distinct samples or whether the same sample was measured repeatedly
☒	☐	The statistical test(s) used AND whether they are one- or two-sided<br>*Only common tests should be described solely by name; describe more complex techniques in the Methods section.*
☐	☒	A description of all covariates tested
☐	☒	A description of any assumptions or corrections, such as tests of normality and adjustment for multiple comparisons
☐	☒	A full description of the statistical parameters including central tendency (e.g. means) or other basic estimates (e.g. regression coefficient) AND variation (e.g. standard deviation) or associated estimates of uncertainty (e.g. confidence intervals)
☒	☐	For null hypothesis testing, the test statistic (e.g. *F*, *t*, *r*) with confidence intervals, effect sizes, degrees of freedom and *P* value noted<br>*Give P values as exact values whenever suitable.*
☒	☐	For Bayesian analysis, information on the choice of priors and Markov chain Monte Carlo settings
☒	☐	For hierarchical and complex designs, identification of the appropriate level for tests and full reporting of outcomes
☒	☐	Estimates of effect sizes (e.g. Cohen's *d*, Pearson's *r*), indicating how they were calculated

Our web collection on statistics for biologists contains articles on many of the points above.

Software and code

Policy information about availability of computer code

Data collection	All data collection for REACT2 is provided with Questback (Spring 2020 installation)
Data analysis	All analysis is performed with R version 4.0.0

For manuscripts utilizing custom algorithms or software that are central to the research but not yet described in published literature, software must be made available to editors and reviewers. We strongly encourage code deposition in a community repository (e.g. GitHub). See the Nature Portfolio guidelines for submitting code & software for further information.

Data

Policy information about availability of data

All manuscripts must include a data availability statement. This statement should provide the following information, where applicable:

- Accession codes, unique identifiers, or web links for publicly available datasets
- A description of any restrictions on data availability
- For clinical datasets or third party data, please ensure that the statement adheres to our policy

The aggregated tables generated during this study are provided as supplemental tables

Field-specific reporting

Please select the one below that is the best fit for your research. If you are not sure, read the appropriate sections before making your selection.

☐ Life sciences ☒ Behavioural & social sciences ☐ Ecological, evolutionary & environmental sciences

For a reference copy of the document with all sections, see nature.com/documents/nr-reporting-summary-flat.pdf

Behavioural & social sciences study design

All studies must disclose on these points even when the disclosure is negative.

Study description	A cross-sectional quantitative survey to explore antibody responses in a population of England
Research sample	In each round of the study we invited a non-overlapping random community sample of adults aged 18 years or over in England, based on the National Health Service general practitioner registrations list.
Sampling strategy	The sample was designed to provide approximately equal numbers of people in each of the 315 lower tier local authority (LTLA) areas in England.Round 6 adopted the design of previous rounds with the addition of a boosted sample of older adults to increase the power to detect whether the risk of becoming a case, being hospitalised or dying from COVID-19 differed between those testing positive and those testing negative on the LFIA following vaccination. Round 6 aimed for a total sample size of 240,000, with inclusion of 70,000 additional people in age groups 55-64 and 65-67 years.
Data collection	Those who registered were sent a Fortress lateral flow immunoassay (LFIA) test kit for SARS-CoV-2 antibody self-testing and asked to perform the test at home, report their test result and upload a photo of the completed test. In addition, they were asked to respond to a questionnaire which included details of COVID-19 vaccination type and date, self-reported co-morbidities and any history of suspected or confirmed COVID-19 infection. Those who had not received a vaccine dose were asked whether they had been invited to take part in the vaccination programme and their response. For those who had not yet been offered a vaccine we asked about their intention to accept.
Timing	For this analysis, participants were recruited in two successive rounds of sampling; round 5 (25 January to 8 February 2021) and round 6 (12 to 25 May 2021).
Data exclusions	We used complete case analysis
Non-participation	We quantify differential response by age, sex, ethnicity and deprivation which are similar to those regularly observed in population surveys and are adjusted for by weighting in the analysis (see supplemental material)
Randomization	N/A

Reporting for specific materials, systems and methods

We require information from authors about some types of materials, experimental systems and methods used in many studies. Here, indicate whether each material, system or method listed is relevant to your study. If you are not sure if a list item applies to your research, read the appropriate section before selecting a response.

Materials & experimental systems

n/a	Involved in the study	
☒	☐	Antibodies
☒	☐	Eukaryotic cell lines
☒	☐	Palaeontology and archaeology
☒	☐	Animals and other organisms
☐	☒	Human research participants
☒	☐	Clinical data
☒	☐	Dual use research of concern

Methods

n/a	Involved in the study	
☒	☐	ChIP-seq
☒	☐	Flow cytometry
☒	☐	MRI-based neuroimaging

Human research participants

Policy information about studies involving human research participants

Population characteristics	See above
Recruitment	We used the Natinoal Health Service Primary Care database to sample adults over the age of 18. Through provision of name and address, personalised invitations were sent to individuals aiming to achieve similar numbers of participants in each of the 315 Lower tier local authorities. Individuals responding to the invitation registered via a website. We quantify differential

	response by age, sex, ethnicity and deprivation which are similar to those regularly observed in population surveys and are adjusted for by weighting in the analysis.
Ethics oversight	We obtained research ethics approval from the South Central-Berkshire B Research Ethics Committee (IRAS ID: 283787), and Medicines and Healthcare products Regulatory Agency approval for use of the LFIA for research purposes only.

Note that full information on the approval of the study protocol must also be provided in the manuscript.
